# Vocal Fold Disorders Classification and Optimization of a Custom Video Laryngoscopy Dataset Through Structural Similarity Index and a Deep Learning-Based Approach

**DOI:** 10.3390/jcm14196899

**Published:** 2025-09-29

**Authors:** Elif Emre, Dilber Cetintas, Muhammed Yildirim, Sadettin Emre

**Affiliations:** 1 Department of Anatomy, Faculty of Medicine, Fırat University, Elazığ 23119, Turkey; eemre@firat.edu.tr; 2 Department of Computer Engineering, Malatya Turgut Ozal University, Malatya 44210, Turkey; dilber.cetintas@ozal.edu.tr; 3 Department of Otorhinolaryngology, Private Elazığ Medikal Hospital, Elazığ 23100, Turkey; sadettinemre23@gmail.com

**Keywords:** artificial intelligence, dysphonia, endoscopic video, laryngoscopy, vocal cord polyps

## Abstract

**Background/Objectives:** Video laryngoscopy is one of the primary methods used by otolaryngologists for detecting and classifying laryngeal lesions. However, the diagnostic process of these images largely relies on clinicians’ visual inspection, which can lead to overlooked small structural changes, delayed diagnosis, and interpretation errors. **Methods:** AI-based approaches are becoming increasingly critical for accelerating early-stage diagnosis and improving reliability. This study proposes a hybrid Convolutional Neural Network (CNN) architecture that eliminates repetitive and clinically insignificant frames from videos, utilizing only meaningful key frames. Video data from healthy individuals, patients with vocal fold nodules, and those with vocal fold polyps were summarized using three different threshold values with the Structural Similarity Index Measure (SSIM). **Results:** The resulting key frames were then classified using a hybrid CNN. Experimental findings demonstrate that selecting an appropriate threshold can significantly reduce the model’s memory usage and processing load while maintaining accuracy. In particular, a threshold value of 0.90 provided richer information content thanks to the selection of a wider variety of frames, resulting in the highest success rate. Fine-tuning the last 20 layers of the MobileNetV2 and Xception backbones, combined with the fusion of extracted features, yielded an overall classification accuracy of 98%. **Conclusions:** The proposed approach provides a mechanism that eliminates unnecessary data and prioritizes only critical information in video-based diagnostic processes, thus helping physicians accelerate diagnostic decisions and reduce memory requirements.

## 1. Introduction

Rapid advances in technology in recent years have made it possible to integrate cameras with many medical devices [1]. This integration has made it possible to visualize difficult-to-reach anatomical regions in detail in clinical practice and to obtain a large number of images of these regions. Laryngoscopy is one of the important methods that benefit from these technological advances. Laryngoscopy is primarily used to obtain detailed information about the structure and function of the larynx, particularly the vocal cords [2]. Laryngoscopy, widely used in the diagnosis of vocal cord pathologies, provides otolaryngologists (ENT) with a rich visual data source for their assessment [3]. This method allows clinicians to directly observe and evaluate lesions in the vocal cords and glottis using laryngoscopy systems [4]. Laryngeal lesions can be benign, such as nodules and polyps, or malignant, such as cancer. These patients often present to outpatient clinics with complaints of dysphonia.

Dysphonia is a clinical term describing deteriorations in the pitch, timbre, intensity, or continuity of the voice, and is most commonly encountered in daily life as hoarseness. The most common structural abnormalities detected in patients suffering from dysphonia are vocal cord nodules and polyps [5]. Vocal cord nodules are one of the most common causes of chronic hoarseness in adults and children among benign laryngeal pathologies. These nodules, which account for approximately 1% of all ear, nose, and throat diseases, are more common in women. They are typically symmetrical, bilateral epithelial thickenings that occur at the junction of the anterior third and middle third of the vocal cords, at the free edge. Nodules are considered reactive changes resulting from incorrect or excessive voice use, rather than an independent disease. The first choice of treatment is vocal hygiene training and voice therapy. Surgery is considered in cases that fail to respond to long-term conservative methods or in cases of fibrotic, chronic nodules [6]. Vocal cord polyps are benign masses that develop as a result of chronic irritation in the larynx. They are most commonly seen in the anterior or middle third of the vocal cords; their surfaces may be vascularized, and they may develop in a pedunculated or sessile form. Unlike nodules, they are usually unilateral. The accumulation of irritation-induced edema in the superficial layer of the lamina propria plays a role in their pathogenesis. Polyps are among the most common benign laryngeal lesions that require surgical intervention. While voice therapy is often sufficient for nodules, it is far from curative for polyps alone [7].

Endoscopic examination is the most crucial step in diagnosing these lesions. Indirect or direct laryngoscopy, using flexible or rigid endoscopes, allows for a detailed assessment of the vocal cord surface and movements. Nodules typically appear as small, symmetrical, and bilateral lesions; polyps are usually unilateral, edematous, and sometimes present with prominent vascular contents. When necessary, vocal cord vibratory properties can also be assessed with stroboscopic examination. Therefore, laryngoscopy is an indispensable method for distinguishing vocal cord polyps and nodules [6,7].

Today, video laryngoscopy not only produces high volumes of video data but also enables access to more detailed diagnostic information. However, this presents a significant challenge: the large number of frames contained in videos necessitates analysis processes that require high processing power and time. Furthermore, a significant portion of consecutive video frames consists of repetitive images that lack distinctive information. This leads to unnecessary consumption of processing resources and reduces the efficiency of the diagnostic process. Therefore, in recent years, researchers in the field of video analysis have chosen to focus solely on key frames to reduce processing load and enable rapid assessment while maintaining diagnostic accuracy. Key frames are diagnostically meaningful and information-dense images selected from the underlying video source, playing a critical role in clinical decision-making [2].

Given subtle early symptoms, approximately 60% of patients seek treatment only when their condition has progressed significantly, thereby missing the optimal time for effective treatment, that is, the early stage of the disease when timely interventions can most effectively slow progression and help maintain patient function. Given the mild early symptoms, approximately 60% of patients seek treatment only when their condition has progressed significantly, thus missing the optimal treatment window (when lesions are smaller) when conservative approaches such as voice therapy and behavioral modification are most effective. In later stages, these methods are often inadequate and may require surgical intervention [8]. Diagnostic aids are critical for taking early precautions and achieving positive outcomes. The lack of endoscopists analyzing laryngoscopic images highlights the need for AI-powered decision support systems. Furthermore, the gag reflex necessitates short-term decision-making, and the presence of lesions invisible to the human eye complicates the work of specialists. Artificial intelligence support can address these challenges, enabling specialists to make more informed decisions.

Laryngoscopy images have not yet been comprehensively examined in AI-based medical imaging research. Ang Meiling et al. aimed to improve the efficiency of diagnostic and decision-making processes by enabling the classification of anatomical regions in electronic laryngoscopy examinations [9]. Tran et al. evaluated laryngoscopic data obtained from 876 patients using various deep learning models; the Xception model achieved 98.90%, 97.36%, and 96.26% accuracy in classifying “no vocal cord,” “normal vocal cord,” and “abnormal vocal cord,” respectively [10]. Additionally, it has been reported that the success rate of this model is higher than that of assistant physicians and comparable to that of specialist physicians.

In studies conducted with machine learning, classification was performed using features such as color, texture, and vascular structure [11,12]. For example, in one study, 4106 images consisting of nine different categories (cyst, nodule, polyp, leukoplakia, papilloma, Reinke’s edema, granuloma, paralysis, and normal cases) were processed with deep neural networks (DNN) and it was reported that the model outperformed clinical experts [13]. Yao et al. developed a ResNet-18 based model for the selection of informative frames from video laryngoscopic images [3]. You et al. achieved 97% accuracy by classifying white light images of vocal cord leukoplakia into six categories using a Siamese network [14]. In another study, the LCDC-AOADL model developed using the Aquila Optimization Algorithm achieved 96.02% accuracy in the classification of larynx cancer [15].

Artificial intelligence applications in laryngoscopy are not limited to classification. Matava et al. developed a system that detects vocal cords and tracheal rings to facilitate real-time recognition of basic anatomical structures for novice physicians [16]. Kim et al. applied segmentation to laryngoscopic images using Mask R-CNN [17]. Studies in laryngoscopy generally focus on classification, object detection, and segmentation. This study aims to select meaningful and informative frames from video laryngoscopic data. This eliminates frames that do not carry similar or distinctive information, allowing processing with less data. This provides significant advantages in terms of both processing power and memory usage. In particular, the high frequency of repetitive and meaningless frames among images acquired per second, depending on the camera’s frame rate (fps), increases the importance of this approach.

Contributions and Novelty:

- While the existing literature mainly focuses on direct analysis methods such as distributions, object variation, and segmentation, this work proposes an approach for automatically selecting meaningful frames from video data.
- In high-frame-rate video laryngoscopy images, unnecessary data processing and storage load are significantly reduced by eliminating frames that are highly similar to each other and are not clinically significant.
- The frame selection method used ensured that only distinctive and information-rich frames were transmitted to the classification and detection stages; thus, despite a significant reduction in the number of data, performance metrics were preserved, and even small but significant positive improvements were observed in some cases.
- An artificial intelligence-based approach to diagnosing vocal cord lesions has been developed, demonstrating that by specifically extracting similar frames from video data, equivalent or higher accuracy results can be achieved using less data.
- The performance of the proposed model was compared with six different pre-trained models accepted in the literature. The two models with the highest performance were used as the base for the proposed model. The performance of the proposed model is better than that of the other models. The proposed model achieved a high accuracy rate of 98%.

The article continues with the Materials and Methods Section. This section includes the dataset developed and the methods used in the study. The Experimental Results Section, which includes the results obtained in the study, is then presented. Finally, the study concludes with the Conclusion Section.

## 2. Materials and Methods

### 2.1. Dataset

In deep learning-based approaches, data is a key factor determining model performance. While increasing the amount of data often positively impacts the model’s learning capacity and generalizability, sufficient data diversity is also necessary for this effect to emerge. Otherwise, the model is likely to memorize the training data (overfitting) and underperform on new data. In video-based medical imaging data, in particular, it is observed that repeated or clinically insignificant frames are frequently present in the dataset due to the high similarity between consecutive frames. Including such frames in the model both reduces the efficiency of the training process and burdens the model with unnecessary information.

To address this disadvantage, this study aimed to select clinically significant and informative frames from video recordings. Thus, the dataset was composed of only distinctive frames. By eliminating unnecessary repetitions and meaningless frames, both training time and memory usage were reduced, and memory usage was optimized.

The dataset used consists of video data recorded with an endoscopic device at a frame rate of 25 frames per second (fps). The videos include cases representing three different clinical conditions: vocally healthy patients, patients with vocal cord nodules, and patients with vocal cord polyps. These diagnoses were made after clinical examination and endoscopic image evaluation by at least two experienced otolaryngologists. In cases of disagreement, a third expert opinion was sought for the final diagnosis. In the presence of polyps or suspicious lesions, surgically removed tissues were confirmed by histopathological examination. Nodule diagnoses were made based on typical clinical and endoscopic features, and conservatively managed cases did not require pathological confirmation. Video recording times varied depending on the patients’ tolerance to the gag reflex. The mean duration was 22.6 s in the control group, 21.7 s in the nodule group, and 21.4 s in the polyp group. This variability in recording length and content provides a natural diversity within the dataset, which is beneficial for training artificial intelligence models by exposing them to heterogeneous data.

The study was approved by the Fırat University Non-Interventional Local Ethics Committee under reference number 2025/09-60 (Meeting Date: 3 July 2025).

**Figure 1:**
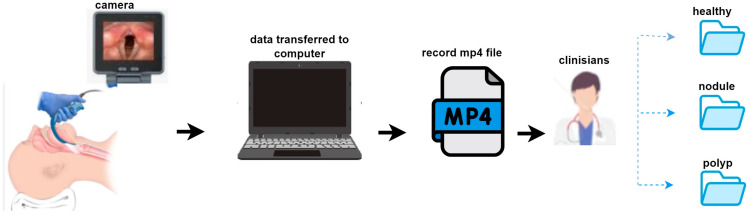
Dataset creation stages.

The dataset consists of video recordings from a total of 51 patients. These recordings comprise three different clinical groups: 20 vocally healthy patients, 15 diagnosed with vocal cord nodules, and 16 diagnosed with vocal cord polyps. Each patient has one video recording. Written informed consent was obtained from all patients prior to video recording, and all videos were anonymized to protect personal data and patient privacy. The data collection process is illustrated in Figure 1. Video recordings were obtained using an endoscopic imaging device and transferred to a computer. All recordings were stored in .mp4 format. Each case was independently assessed by two otolaryngologists, and in situations where disagreement occurred, a third senior otolaryngologist was consulted to reach consensus.

All lesions were diagnosed by experienced otolaryngologists using laryngoscopic examination. Each case was independently evaluated by two otolaryngologists, and in cases of disagreement, a third senior otolaryngologist was consulted to reach consensus. Histopathological confirmation was obtained only in patients who underwent surgical excision, while conservatively managed patients were diagnosed based on clinical findings without biopsy.

### 2.2. Structural Similarity Index Measure (SSIM)

Video summarization is a compact representation of videos, enabling the selective presentation of important events occurring within the content [18]. This summarization process allows users to access meaningful information more quickly. In this study, the video summarization approach was applied to visual frames in the medical field. In clinical applications, effectively utilizing raw diagnostic video data is challenging because processing, annotating, and reviewing such data are time-consuming [19]. Therefore, key frames were extracted by comparing correlation and structural features between frames. SSIM, formulated by Wang et al. [20], was used for measuring structural similarity. SSIM quantitatively measures the perceived visual similarity between two images and compares components meaningful to the human visual system, such as brightness, contrast, and structural content, rather than solely evaluating pixel-based differences. SSIM ranges from 0 to 1, with a value of 1 indicating that the two images are structurally identical, while a value of 0 indicates they are structurally dissimilar.

In clinical video summarization applications, SSIM is used to determine the structural similarity between frames, and representative frames with high similarity are selected as keyframes within each set. This minimizes information loss in the video while presenting the summarized content to the user in a more compact and meaningful format. In a medical summary study on hysteroscopy, various summarization techniques were evaluated, and a hybrid model that combined motion, contrast, texture, and clarity of curvature was found to have higher success [21].

### 2.3. Keyframe Extraction

**Figure 2:**
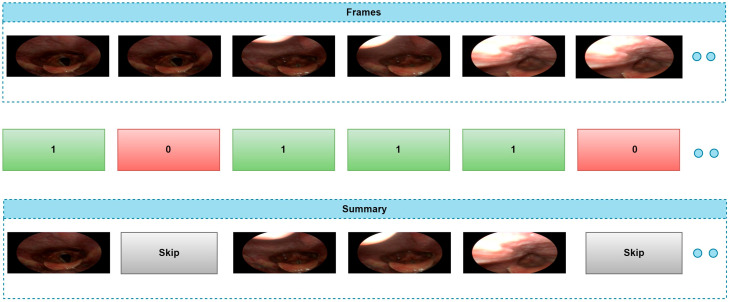
Frame Extraction.

Keyframe extraction is considered a fundamental preprocessing step in video analysis [22]. The goal of this step is to select meaningful and information-rich frames from all frames of a video, reducing data volume and simplifying subsequent analysis processes. However, this task is particularly challenging, especially for medical image data, because the number of frames exhibiting similar structural features within the same video is relatively high, making it difficult to capture frames containing anomalous regions or pathological content accurately. In this study, keyframe extraction is performed by calculating a measure of structural similarity between consecutive frames. A similarity value is obtained using SSIM for each pair of consecutive frames, and this value is compared to a predetermined threshold. Frames with SSIM values below the threshold are considered content-distinct and potentially essential and are selected as keyframes. This process is repeated until all frames in the video have been processed, thereby selecting only frames containing meaningful information for summarization. Keyframe extraction is illustrated in Figure 2.

### 2.4. Proposed Methodology

The current study presents an approach that integrates video summarization and hybrid CNN models. CNNs, thanks to their powerful learning capabilities, can achieve high-accuracy results on image data. The SSIM method is used to measure image similarity. This method compares the similarity score obtained by comparing two images with a predetermined threshold value to determine whether the relevant image should be selected as a keyframe. The primary objective of the designed model is to minimize processing costs and memory usage while maintaining accurate performance by working with more meaningful and representative data, rather than large amounts of repetitive data.

**Figure 3:**
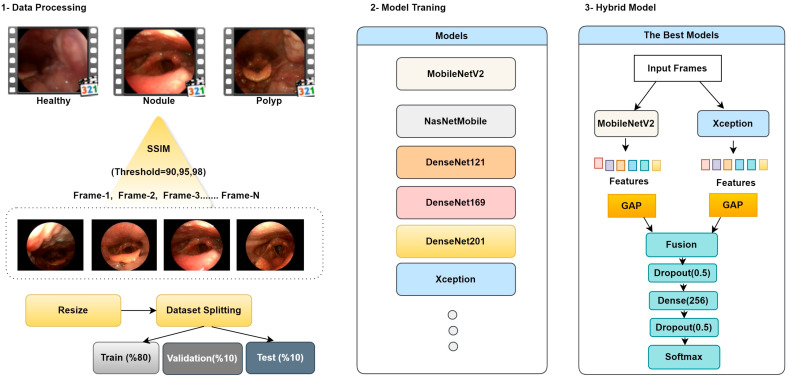
Proposed Methodology.

In the feature extraction process, the MobileNetV2 architecture was used to extract fine details and local edge information. In contrast, the Xception architecture, thanks to its deeper structure, captured global context and high-level semantic information. Feature maps obtained from different architectures were combined using the concatenation method to create a richer and more distinctive feature representation. Combining feature maps obtained from CNN layers enables more efficient use of information and increases model accuracy. The general workflow and structural components of the proposed method are presented in Figure 3.

**Step 1: Data Processing and Keyframe Extraction;** In this phase, videos from healthy individuals, patients with vocal cord nodules, and patients with vocal cord polyps in the dataset are summarized and segmented into frames using the SSIM technique. Threshold values for the summarization process were set as 90%, 95%, and 98%. Due to the nature of medical images and to preserve existing lesion areas, lower threshold values were not chosen. Based on the specified threshold values, for example, when the threshold is set to 90%, frames with SSIM values below 90% are considered content-different and are saved as keyframes. Conversely, frames with SSIM values above 90% are eliminated from the summarization process because they are similar content captured sequentially, depending on the number of frames per second. This removes repetitive frames with minimal informational value, ensuring that more meaningful and representative images are included in the processing. Table 1 provides a threshold comparison table.

As the threshold value increases, the number of extracted keyframes increases steadily. Lower threshold values allow for the selection of fewer but more distinct frames, while higher threshold values minimize detail loss but increase the redundancy rate. Because it is crucial to avoid losing lesion details in medical images, a threshold value of 0.90 was not used in this study. Obtaining fewer frames results in a smaller but potentially more diverse dataset for training the model.

The extracted keyframes were resized to the appropriate dimensions for use as input to deep learning models. To ensure a balanced use of the model in the training and validation processes, the data were then randomly split into two subsets: 80% for training and 20% for validation.

**Step 2: Model Training;** In this step, the impact of keyframe extraction on model performance was investigated using various deep learning models. Each model was trained separately using input keyframes generated from keyframe sets obtained using thresholds of 90%, 95%, and 98%. To ensure comparable results across models, all training processes were conducted under the same data separation, hyperparameters, training time, learning rate, and hardware conditions. This ensured that performance differences were solely due to the threshold values and model architectures used in keyframe extraction.

**Step 3: Hybrid Model;** In this study, comprehensive ablation experiments were conducted using three different threshold values and the MobileNetV2 [23], NASNetMobile [24], DenseNet (121/169/201) [25], and Xception [26] architectures. Based on the obtained results, the most appropriate threshold value and the most successful individual models were determined, and a hybrid architecture was designed based on these findings. In the hybrid architecture, pre-trained weights were initially frozen; then, during the fine-tuning phase, the last 20 layers of each vertebra were opened for training, enabling the learning of higher-level representations. The resulting features were combined and transferred to fully connected layers; a dropout layer was placed between layers to reduce overfitting, and an early stopping strategy was implemented. Finally, the architecture is completed with a softmax-based output layer for multi-class discrimination.

### 2.5. Performance Metrics

Measures such as sensitivity, recall, F1-Score, and accuracy are widely used to evaluate classification performance in ablation studies. These metrics are calculated using true-positive (TP), true-negative (TN), false-positive (FP), and false-negative (FN) values, which quantitatively reflect the relationship between the model’s actual classification output and the predicted results [27]. Each metric takes normalized values between 0 and 1, and the formulations used in the study are presented in Equations (1)–(5).

(1) $$\mathrm{Accuracy}=\frac{TP+TN}{(TP+TN+FP+FN}$$

(2) $$\mathrm{Precision}=\frac{TP}{TP+FN}$$

(3) $$\mathrm{Recall}=\frac{TP}{TP+FP}$$

(4) $$\mathrm{F1 Score}=\frac{2TP}{2TP+FP+FN}$$

(5) $$\mathrm{MCC}=\frac{\left(TP\cdot TN\right)-\left(FP\cdot FN\right)}{\sqrt{\left(TP+FP\right)\left(TP+FN\right)\left(TN+FP\right)\left(TN+FN\right)}}$$

## 3. Experimental Results

This study presents a hybrid deep learning approach based on keyframe extraction for analyzing medical videos. All experiments were conducted on Google Colab Pro using the TPU accelerator, and the findings were evaluated within the framework of the metrics presented in the “Performance Metrics” subsection. Considering the data dependency of deep learning methods, it was observed that meaningful and informative examples increase model performance; however, repetitive and uninformative frames increase computational load and cost. Therefore, redundancy was reduced through keyframe selection to increase data efficiency.

In the first phase of the experimental design, a fully connected layer and a softmax classifier were added to the pre-trained frozen backbones to determine the effectiveness of the candidate architectures. The results of these screening experiments are reported in Table 2. The training process for all models was conducted with fixed hyperparameters: optimizer: Adam, learning rate: 0.001, batch size: 32, and epochs: 30. To reduce overfitting, a dropout layer was placed before the classifier, and training was supported by early stopping strategies.

When the results in Table 2 are examined, it is observed that all models exhibit similar performance under different SSIM threshold values (0.90, 0.95, 0.98), although the rankings change with minor differences. The MobileNetV2 and Xception models stood out with high accuracy and strong MCC scores at all three threshold values. The DenseNet family also demonstrated similarly stable performance. In contrast, NasNetMobile performed less well than the other models.

**Figure 4:**
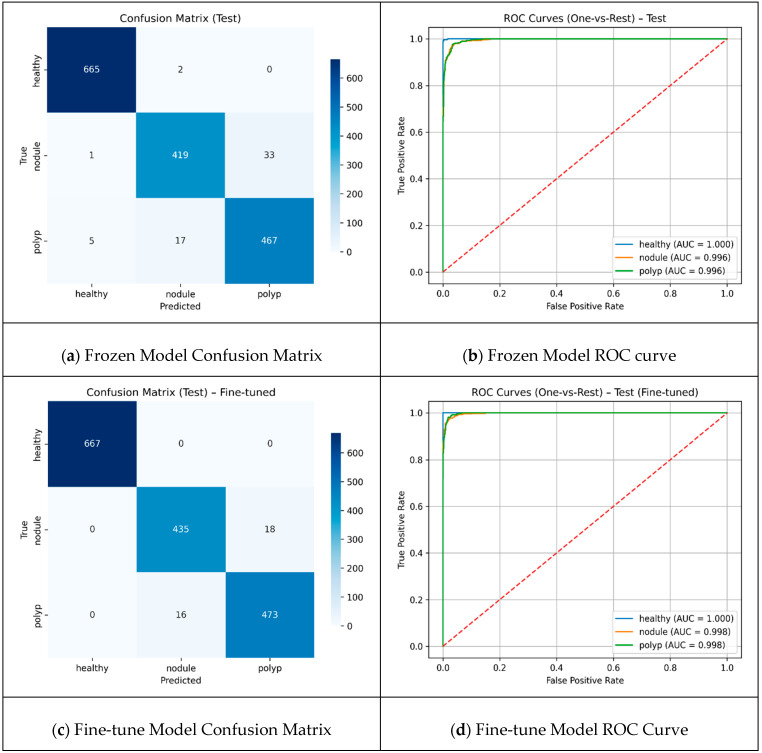
Scenario results (**a**) Frozen Model Confusion Matrix (**b**) Frozen Model ROC curve (**c**) Fine-tune Model Confusion Matrix (**d**) Fine-tune Model ROC Curve.

A threshold value of 90 resulted in the selection of more distinct frames as keyframes, allowing the models to be trained with more diverse data. The results reached their highest level, with MobileNetV2 and Xception in particular standing out, achieving approximately 91% accuracy and high MCC values. Although more frames were obtained at 0.98, the repetitive data limited the model’s discriminative learning capacity and reduced performance metrics. In the hybrid model design, the keyframes extracted with the threshold value that yielded the best performance from the ablation experiments were used as input for feature extraction from the two most successful backbones. These two backbones were evaluated in two different scenarios: transfer learning, where only the top classifiers were trained with frozen layers, and fine-tuning, where the last layers were retrained. The results of both scenarios are reported using confusion matrices and ROC curves to provide a holistic illustration of the classification performance, with detailed visualizations presented in Figure 4.

The comparison presented in Figure 4 reveals that the total number of misclassifications decreased by approximately 41%, from 58 to 34, and the fine-tuning method demonstrated superior performance. The healthy class was identified with high accuracy by both approaches, and error-free classification was achieved in this class with fine-tuning. The number of correct classifications for the nodule class increased from 419 to 435, demonstrating a significant improvement in the clinically critical class. The majority of errors were concentrated at the “nodule-polyp” boundary. This finding suggests that fine-tuning enhances the discrimination between classes; however, specific challenges remain in distinguishing morphologically similar lesions. The Performances of frozen and fine-tuned models are presented in Table 3.

The fine-tuned model demonstrates a consistent and balanced class-wide superiority over the frozen model. While accuracy was 96% in the frozen case, it increased to 98% in the scenario where the last 20 layers were trained with fine-tuning. The already very high performance in the healthy class becomes completely flawless with fine-tuning. The most striking improvement is in the nodule class; the recall rate increases from 92% to 96%, which indicates fewer missed nodules. This translates into fewer missed diagnoses, a critical factor for early treatment decisions and improving clinical reliability of the model. For the polyp, both precision and recall show small but consistent increases, with F1 reaching from 0.94 to 0.97. The results demonstrate that fine-tuning can further generalize overall performance. Cross-Validation Fold Metrics are presented in Table 4.

Table 4 shows that the model achieved consistently high precision, recall, and F1-scores across all classes, with particularly strong performance for the healthy class (F1 = 0.9964 ± 0.0010). While the nodule and polyp classes yielded slightly lower values (F1 ≈ 0.966–0.970), the overall results indicate robust classification performance.

The proposed approach reduces the amount of data to be processed by selecting only clinically significant frames from video laryngoscopy images, accelerating the diagnostic process. SSIM-based frame selection eliminates similar and repetitive frames, reducing the model’s computational load and ensuring more efficient memory usage. The hybrid architecture, created by combining deep features from MobileNetV2 and Xception, demonstrated high classification accuracy (98%). This approach offers an innovative alternative to direct analysis methods in the existing literature, achieving high accuracy with a small yet information-rich dataset. However, the study also has some limitations. Using fixed SSIM threshold values may not adequately adapt to variations across different patient groups. Furthermore, the dataset used only includes three classes (healthy, nodule, and polyp), and the model’s generalizability to a broader range of lesion types has not been tested. Therefore, future studies with different frame selection strategies and larger datasets will enable a more comprehensive evaluation of the method for clinical applications.

## 4. Conclusions

This study presents a hybrid CNN-based approach that eliminates repetitive and clinically insignificant frames to improve accuracy and efficiency in the analysis of video laryngoscopy images. Using the Structural Similarity Index (SSIM) method with different threshold values, only key frames with high diagnostic value are automatically selected, reducing data volume while maintaining and even improving model performance. Experimental findings demonstrate that a threshold of 0.90 provides the most efficient results in terms of frame diversity and classification accuracy. The hybrid architecture, created by combining deep features derived from MobileNetV2 and Xception architectures, achieved a high classification accuracy of 98%. This approach not only reduces computational and storage costs but also directly contributes to clinical decision-making by accelerating the diagnostic process. An AI-based tool is presented that reduces clinicians’ workload and enables them to make more reliable and rapid decisions, particularly in cases of vocal cord lesions, where early diagnosis is crucial.

The model currently includes only three categories (healthy, nodule, and polyp). Other laryngeal pathologies such as cysts, leukoplakia, papillomas, granulomas, and particularly early-stage laryngeal cancers were not included in this study. Therefore, the model is not yet sufficient for real-world diagnostic environments where clinicians must distinguish across a broader spectrum of benign and malignant conditions. This study was limited to benign vocal fold lesions, specifically nodules and polyps. These entities were chosen because of their high prevalence in routine clinical practice, which provided a stable and balanced dataset to develop and validate the proposed machine learning methodology. Malignant lesions were intentionally excluded, as their diagnosis and management require urgent oncological intervention and involve distinct clinical pathways that were beyond the scope of this research.

A limitation of this study is the relatively small number of patients. Although the large number of frames and extracted keyframes provided sufficient data for deep learning, future studies with larger and more diverse patient cohorts are needed to enhance the statistical robustness and clinical generalizability of the results. Another limitation is that only benign lesions were considered. Histopathological confirmation was available for surgical cases, whereas conservatively managed patients were classified by expert consensus, which may introduce some diagnostic bias. Moreover, functional voice assessments such as GRBAS or CAPE-V were not included, as they are not routinely used for differentiating nodules from polyps, though future studies may benefit from correlating anatomical findings with voice quality measures.

While natural variability in video quality may enhance generalization, it may also reduce reliability in extreme cases. As a future direction, we propose dedicated robustness testing under controlled degradations (e.g., simulated noise, blur, or lighting variations) and multi-center validation to ensure that the model maintains consistent performance across diverse clinical environments.

Future studies aim to test the method with larger and more diverse patient groups, increase its generalizability to include different lesion types, and investigate adaptive frame selection strategies. In addition, future work will focus on practical clinical applicability, such as integrating the system into real-time endoscopic procedures to assist clinicians during lesion detection, and conducting multi-center validation across different hospitals and devices to confirm robustness and facilitate broader clinical adoption.

## Figures and Tables

**Table 1:** Keyframe Numbers Obtained According to SSIM Threshold Values.

Threshold	Healthy	Nodule	Polyp	Total
**0.90**	9412	6412	7099	22,923
**0.95**	10,295	7841	8231	26,367
**0.97**	10,702	8086	8457	27,245
**0.98**	10,961	8175	8532	27,668
**0.99**	11,279	8237	8616	28,132

**Table 2:** Macro Avg. Results of Models According to Keyframes Obtained from Different SSIM Threshold Values.

Threshold	Method	Precision	Recall	F1-Score	Accuracy	MCC
**90**	MobileNetV2	90.37	90.17	90.23	90.85	86.20
	NasNetMobile	84.89	84.73	84.80	86.23	79.00
	DenseNet121	85.57	8540	85.47	86.94	80.01
	DenseNet169	88.28	88.18	88.13	89.34	83.90
	DenseNet201	88.78	88.70	88.55	89.65	84.40
	**Xception**	90.06	89.66	89.81	90.71	85.90
95	MobileNetV2	90.37	90.17	90.23	90.85	86.20
	NasNetMobile	85.73	85.63	85.63	86.62	79.80
	DenseNet121	86.51	86.24	86.30	87.34	80.90
	DenseNet169	88.68	88.60	88.64	89.46	84.10
	DenseNet201	89.15	89.06	89.10	89.85	84.70
	**Xception**	89.32	89.16	89.13	89.74	84.60
98	MobileNetV2	88.95	88.64	88.74	89.52	84.20
	NasNetMobile	84.58	84.33	84.34	85.50	78.10
	DenseNet121	85.95	85.61	85.62	86.79	80.10
	DenseNet169	87.73	87.55	87.53	88.48	82.60
	DenseNet201	88.58	88.34	88.37	89.26	83.80
	**Xception**	88.68	88.60	88.64	89.46	84.10

**Table 3:** Performances of frozen and fine-tuned models.

**Frozen**				
	Precision	Recall	F1-score	Accuracy
**Healthy**	0.99	1.00	0.99	0.96
**Nodule**	0.96	0.92	0.94	
**Polyp**	0.93	0.96	0.94	
**Fine-Tune (Last 20 Layers)**				
**Healthy**	1.00	1.00	1.00	0.98
**Nodule**	0.96	0.96	0.96	
**Polyp**	0.96	0.97	0.97	

**Table 4:** Cross-Validation Fold Metrics.

	Precision (Mean ± Std)	Recall (Mean ± Std)	F1-Score (Mean ± Std)	Support (avg)
healthy	0.9958 ± 0.0022	0.9970 ± 0.0014	0.9964 ± 0.0010	~1317.6
nodule	0.9692 ± 0.0082	0.9630 ± 0.0092	0.9660 ± 0.0032	~897.6
polyp	0.9676 ± 0.0088	0.9714 ± 0.0079	0.9695 ± 0.0023	~993.8

## Data Availability

The data set will be shared upon the researchers’ request.
